# Novel Approach for the Recognition and Prediction of Multi-Function Radar Behaviours Based on Predictive State Representations

**DOI:** 10.3390/s17030632

**Published:** 2017-03-19

**Authors:** Jian Ou, Yongguang Chen, Feng Zhao, Jin Liu, Shunping Xiao

**Affiliations:** 1 Key Laboratory of Complex Electromagnetic Environment Effects on Electronics and Information System, National University of Defence Technology, Changsha 410073, China; zhfbee@tom.com (F.Z.); liujin@nudt.edu.cn (J.L.); qwertmingx@tom.com (S.X.); 2 Beijing Institute of Tracking & Telecommunications Technology, Beijing 100094, China; ygchen@netease.com

**Keywords:** predictive state representation, multi-function radar, signal prediction, operating mode recognition

## Abstract

The extensive applications of multi-function radars (MFRs) have presented a great challenge to the technologies of radar countermeasures (RCMs) and electronic intelligence (ELINT). The recently proposed cognitive electronic warfare (CEW) provides a good solution, whose crux is to perceive present and future MFR behaviours, including the operating modes, waveform parameters, scheduling schemes, etc. Due to the variety and complexity of MFR waveforms, the existing approaches have the drawbacks of inefficiency and weak practicability in prediction. A novel method for MFR behaviour recognition and prediction is proposed based on predictive state representation (PSR). With the proposed approach, operating modes of MFR are recognized by accumulating the predictive states, instead of using fixed transition probabilities that are unavailable in the battlefield. It helps to reduce the dependence of MFR on prior information. And MFR signals can be quickly predicted by iteratively using the predicted observation, avoiding the very large computation brought by the uncertainty of future observations. Simulations with a hypothetical MFR signal sequence in a typical scenario are presented, showing that the proposed methods perform well and efficiently, which attests to their validity.

## 1. Introduction

With the development and application of the active electronically scanned array (AESA), multi-function radar (MFR) [1,2] has become a sensing system with multiple operating modes and multiple tasks that are of great intelligence, flexibility, and adaptability. The common technologies of radar countermeasures (RCMs) and electronic intelligence (ELINT) [3,4] do not perform well enough when dealing with MFRs, especially with the ones taking electronic counter-countermeasures [5,6,7,8]. However, the recently-proposed cognitive electronic warfare (CEW) [9,10,11] is able to take countermeasures according to the electromagnetic environment of the battlefield, possibly achieving better performance. There are several studies on the topic of radar emitter recognition [12,13,14,15,16,17], while the focus mostly goes to the recognition of the emitter model or individual. Although this intelligence information is valuable for jammers, the key factor that affects the jamming decision is the current and future behaviours of radar, including the operating modes, waveform parameters, scheduling schemes, etc., which are exactly what the CEW concentrates on. Therefore, it is urgent to determine how to identify and predict these behaviours of MFRs accurately in CEW.

MFR operating mode recognition [14,15] is to classify the MFR modes by extracting the typical characteristics from intercepted MFR signals, which is an important measure in analysing the MFR behaviours. In 2003, Visnevski developed a hierarchical radar model [18] and viewed the MFR signal sequence as a discrete dynamic stochastic process. Because of the radar resourses scheduling schemes [19,20], MFR signals almost certainly contains some potentially regular characteristics. Therefore, the Markov property of the sequence will be the major basis for recognition and prediction. Based on the hierarchical structure, Visnevski and Krishnamurthy utilized the syntactic model to recognize the radar modes [21,22], but their approach is limited by the premise that the multiple layers must be divided accurately. Visnevski modelled MFR based on the hidden Markov model (HMM) [22] and used the grid-filter to estimate the posterior probability of operating modes. The grid-filter-based method performs well; however, the mode transition probabilities used in the filter are actually dynamically changing. These probabilities are almost unavailable in application, especially considering the game relationship between MFRs and ELINT systems in EW [23].

As for the prediction of MFR signals, few studies are reported in public literature. Considering that the MFR signals intercepted by ELINT systems can be used as training samples, the data-driven predicting methods [24,25,26,27] should be proper alternatives. Predictive state representation (PSR) [28] is a data-driven dynamic system model, which has the advantages of being more generic, more powerful in representation, and easier to learn. Wolfe proposed the multi-mode PSR (MMPSR) which is able to model uncontrolled dynamical systems that switch between several modes of operation [29], providing new thoughts for solving this problem. Although PSR models can be naturally used as a predictor [30] to calculate the exact prediction probabilities, the normal algorithm that directly makes use of the property of linear PSR is also to be improved in computational complexity, in particular for the high-dimensioned PSR model of MFR.

Generally speaking, because of the complexity and variety of MFR signals, the drawbacks of existing methods make them difficult to apply practically. In [31], we constructed the framework of the PSR-based MFR model, which significantly outperforms HMM in radar operating mode recognition. However, it is also based on a grid-filter and cannot be used for predicting MFR signals. In this paper, we attempt to address the drawbacks in MFR behaviour analysis with the PSR-based framework. Firstly, regarding the difficulty in obtaining prior information, a novel recognition method for the MFR operating mode is studied without the help of transition probabilities. Then, regarding the problem of the high computational complexity of the normal algorithm, the fast prediction algorithm for MFR signals is studied. Finally, comparing with the common methods, simulation results are presented to attest to the validity of the proposed approaches.

## 2. PSR-Based Framework of MFR

According to the PSR-based framework of MFR we constructed in [31], the MFR is modelled by combining PSR with a hierarchical structure, taking the advantages of PSR in dynamic system representation and characteristic extraction. The model is pre-trained by utilizing the MFR signals intercepted by the ELINT system. When used in MFR behaviour recognition, the PSR-based framework is attested to have better performance than HMM, in particular for signal sequences with unknown regular characteristics, regardless of whether the radar modes change frequently. The framework is briefly described below.

### 2.1. PSR and System-Dynamics Matrix

Since reconnaissance equipment intercepts radar signals passively, the radar signal sequence is an uncontrolled stochastic process from the point of view of ELINT systems. The PSR model for an uncontrolled system can be represented as a four-tuple <*o*, *h*, *e*, *p*(*e*|*h*)>:

- *o*—observation, and the finite discrete set constituted by all observations is called the observation space, denoted as *o* ∈ *O*;
- *h—*history, which is an observation sequence from the initial time to the current time, *h* = *o*_1_*o*_2_…*o_t_*;
- *e*—event, which is an observation following the history, *e* = *o_t+_*_1_*o_t+_*_2_…. For linear PSR, if any event probability can be expressed by a linear combination of the probabilities of events in a set, the elements in the set are called core events, *Q* = {*q*_1_, *q*_2_, …, *q*_|*Q*|_};
- *p*(*e*|*h*)—the probability of event *e* under the condition of a given history *h*.

In mathematics, a controlled or uncontrolled system can be described by the system-dynamics matrix [30]. The system-dynamics matrix was introduced by Singh in 2004. The partially-observable Markov decision process (POMDP), HMM, and PSR can all be derived readily from it [32]. Constructing a matrix with lines and rows corresponding to all events and histories, the element in the *i*th row and *j*th column of the matrix represents the probability of event *e_j_* under the condition of history *h_i_*, yielding the system-dynamics matrix.

(1) $$D=\left[\begin{array}{cccc} p(e_{1}|h_{0}=\phi ) & \cdots & p(e_{j}|h_{0}=\phi ) & \cdots \\ \vdots & \ddots & \vdots & \cdots \\ p(e_{1}|h_{i}) & \cdots & p(e_{j}|h_{i}) & \cdots \\ \vdots & \vdots & \vdots & \ddots \end{array}\right].$$

The zero-length or initial history ϕ is included in the matrix.

### 2.2. PSR Model for MFR Hierarchical Structure

**Figure 1:**
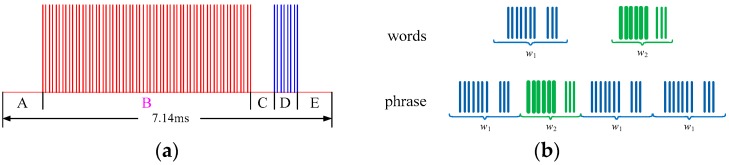
The hierarchical structure of MFR signals. (**a**) A radar word structure of the “Mercury” radar; and (**b**) layered signal structure of a hierarchical MFR example.

To analyse MFR signals more effectively, a commonly used layered model for MFR signals, developed by Haykin and Visnevski [18], is introduced. They put forward the definition of radar “words” as static or dynamically varying groups of pulses that MFRs emit in different states, which can be viewed as fundamental building blocks of the MFR signals. Take, for example, the “Mercury” airborne MFR given in [18,21,22], whose radar word structure is shown in Figure 1a. Its words are all of equal length of 7.14 ms and made up of five sections A–E. Sections A, C, and E are dead time of known duration. Section B is a fixed pulse repetition interval (PRI) pulse-Doppler sequence, and D is a scheduled PRI synchronization burst with 12 pulses. A radar “phrase” is made up of a finite number of words in a fixed order, such as the phrase ‘*w*_1_*w*_2_*w*_1_*w*_1_‘ in Figure 1b.

Below, a PSR model for an MFR word sequence is constructed by combining the hierarchical structure with the PSR concepts:

*W* is the finite set containing all words. If each phrase is concatenated with *n* words, the observation *o_t_* at any time *t* is an *n*-word short sequence, and the observation space *O* = *W^n^*. Event *e* is an observation sequence after current time, such as *o_t_*_+1_*o_t_*_+2_*o_t_*_+3_.

The vector of probabilities corresponding to *Q* = {*q*_1_, *q*_2_, …, *q*_|*Q*|_} is:

(2) $$p(Q|h)={\left[p(q_{1}|h),p(q_{2}|h),\cdots ,p(q_{\left|Q\right|}|h)\right]}^{T}.$$

According to the definition of *Q*, the probability of any observation can be expressed by a linear combination of **p**(*Q*|*h*). Thus, there exists a column vector of weights **m***_o_* such that:

(3) $$p(o|h)=p^{T}(Q|h)m_{o},$$

for each possible observation *o*. Set $M_{o}=[m_{oq_{1}},m_{oq_{2}},\cdots ,m_{oq_{\left|Q\right|}}]$. When a new observation *o* is received, p(*Q*|*h*) is updated as:

(4) $$p^{T}(Q|ho)=\frac{p^{T}(Q|h)M_{o}}{p^{T}(Q|h)m_{o}}.$$

We studied based on the hierarchical structure and focused on the radar word sequence after signal sorting and word extracting for a single radar.

### 2.3. Process for MFR Behaviours

**Figure 2:**
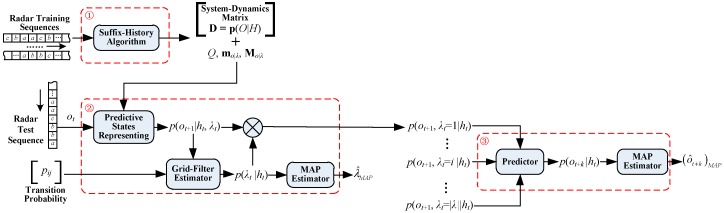
The process for MFR behaviour recognition and prediction based on PSR.

Based on PSR and the hierarchical structure of MFR signals, the framework of recognition and prediction for MFR behaviours is presented in Figure 2.

There are three steps in the total process, as shown in the dashed boxes in Figure 2, respectively:

(1) Modelling and training for the PSR-based MFR models. The system-dynamics matrix for each operating mode will be obtained via suffix-history algorithm [33], discovering the sets of core events *Q*, and yielding the model parameters, such as **m***_o__|λ_* and **M***_o__|λ_*.
(2) Posteriori probability distribution estimation for MFR operating modes. The modes in the input test sequence are identified, and the distribution is calculated via the grid-filter estimator.
(3) Multi-step prediction for MFR signal sequence. With the fusion of prediction results for each mode, the prediction probability distribution for all observations is calculated, from which the prediction results are estimated under the MAP criterion.

For the PSR model in each mode *λ* and under the condition of history *h*:

(5) $$p\left(o|h,\lambda \right)=p^{T}(Q|h,\lambda )\cdot m_{o|\lambda }.$$

(6) $$p\left(oQ|h,\lambda \right)=p^{T}(Q|h,\lambda )\cdot M_{o|\lambda }.$$

where $M_{o|\lambda }=[m_{oq_{1}|\lambda },m_{oq_{2}|\lambda },\cdots ,m_{oq_{\left|Q\right|}|\lambda }]$. Therefore, the probability vector of core events will be updated as:

(7) $$p^{T}\left(Q|ho,\lambda \right)=\frac{p^{T}\left(Q|h,\lambda \right)M_{o|\lambda }}{p^{T}\left(Q|h,\lambda \right)m_{o|\lambda }}.$$

The modelling and pre-training of PSR have been completed. Then, based on the pre-trained PSR models, we focus on Steps (2) and (3) in Figure 2 and attempt to improve the existing methods for recognition and prediction.

## 3. Novel Algorithms for MFR Behaviour Recognition and Prediction

### 3.1. Recognition for Operating Modes

The key to recognition for MFR modes is to estimate the posterior probability of every mode. Although the grid-filter-based methods in [22,34] perform well, the transition probabilities used are dynamical and unlikely to be obtained, which means their values should not be fixed. Below, a novel algorithm is proposed for maximum a posteriori (MAP) estimation of MFR modes by judging the mode transitions according to the accumulated predictive states, instead of utilizing the transition probabilities.

The improved PSR-based algorithm is a two-step process: firstly, estimating the distribution in each mode for the next observation *p*(*o_t_*_+1_|*h_t_*, *λ_t_* = *i*); secondly, estimating the posteriori distribution of the corresponding operating mode *p*(*λ_t_* = *i*|*h_t_*). Firstly, the process for the former is iterative, as follows:

**Step 1:** Initialization: At the initial time *t* = 0 (*h*_0_ = ϕ):

(8) $$p(Q|\phi ,\lambda_{t})={\left[p(q_{1}|\phi ,\lambda_{t}),p(q_{2}|\phi ,\lambda_{t}),\cdots ,p(q_{\left|Q\right|}|\phi ,\lambda_{t})\right]}^{T}.$$

It has been proved in [35] that different initial states have no effect on the PSR model parameters. Thus, the elements in **p**(*Q*|ϕ, *λ_t_*) can be simply replaced by the occurrence frequency of each core event, yielding in the modelling step via the suffix-history algorithm.

For the first observation *o*_1_:

(9) $$p\left(o_{1}|\phi ,\lambda_{t}\right)=p^{T}(Q|\phi ,\lambda_{t})\cdot m_{o_{1}|\lambda_{t}}.$$

**Step 2:** Iteration: According to Equation (7), **p**(*Q*|*h*, *λ_t_*) is to be updated by:

(10) $$p^{T}(Q|h_{t},\lambda_{t})=p^{T}(Q|h_{t-1}o_{t},\lambda_{t})=\frac{p^{T}(Q|h_{t-1},\lambda_{t})M_{o_{t}|\lambda_{t}}}{p^{T}(Q|h_{t-1},\lambda_{t})m_{o_{t}|\lambda_{t}}},$$

yielding:

(11) $$p(o_{t+1}|h_{t},\lambda_{t})=p^{T}(Q|h_{t},\lambda_{t})m_{o_{t+1}|\lambda_{t}},\;o_{t+1}\in O.$$

The vector $m_{o_{t+1}|\lambda_{t}}$ is used to predict the observation by weighting the prediction probability of all core events in mode *λ_t_*, which reflects the nature of the PSR model of expressing the current state utilizing the probability distribution in the future. Then, the results of Equation (11) are used to estimate the mode posterior distribution *p*(*λ_t_* = *i*|*h_t_*).

Since the sum of the posterior probabilities of all operating modes is 1, they can be obtained just by calculating and normalizing their ratio. Considering that in general battlefield scenario, the relative position between MFR and single target will not suddenly change, resulting in that the MFR mode remains unchanged within a short time [1,2]. Therefore, the crux of recognition is to judge the transitions of MFR modes. With this assumption, it is apparent that *λ_t_*_−1_ = *λ_t_* for any operating mode, yielding:

(12) $$p(\lambda_{t}=i|h_{t})=p(\lambda_{t}=i|h_{t-1}o_{t})=\frac{p(o_{t}|h_{t-1},\lambda_{t-1}=i)}{p(o_{t}|h_{t-1})}\cdot p(\lambda_{t-1}=i|h_{t-1}).$$

Equation (12) has a recursive form. Thus, recursion to the initial moment yields:

(13) $$p(\lambda_{t}=i|h_{t})=\frac{\prod_{\tau =1}^{t}p(o_{\tau }|h_{\tau -1},\lambda_{\tau -1}=i)}{\prod_{\tau =1}^{t}p(o_{\tau }|h_{\tau -1})}\cdot p(\lambda_{\tau -1}=i|\phi ),$$

where *p*(*o_τ_*|*h_τ_*_−1_, *λ_τ_*_−1_ = *i*) for each *τ* is calculated by solving Equation (11); the denominator can be offset when normalized due to its independence from *λ_t_*. If the initial probabilities of mode obeys the uniform distribution *p*(*λ_t_* = *i*|ϕ) = 1/|*λ*|, the posteriori probability can be derived by normalizing Equation (13) as:

(14) $$p(\lambda_{t}=i|h_{t})=\frac{\prod_{\tau =1}^{t}p(o_{\tau }|h_{\tau -1},\lambda_{\tau -1}=i)}{\sum_{i=1}^{\left|\lambda \right|}\prod_{\tau =1}^{t}p(o_{\tau }|h_{\tau -1},\lambda_{\tau -1}=i)}.$$

It is shown in Equation (14) that, actually, all predictive states *p*(*o_τ_*|*h_τ_*_−1_, *λ_τ_*_−1_ = *i*) for the entire history are accumulated with the assumption that the modes remain the same within a short time. Considering the Markov property of an MFR signal sequence, the probabilities of states are only related to the latest several states. Thus, only a certain number of probabilities need to be accumulated to judge the mode transitions, and if the number is *r*:

(15) $$p(\lambda_{t}=i|h_{t})\approx \frac{\prod_{\tau =t-r}^{t}p(o_{\tau }|h_{\tau -1},\lambda_{\tau -1}=i)}{\sum_{i=1}^{\left|\lambda \right|}\prod_{\tau =t-r}^{t}p(o_{\tau }|h_{\tau -1},\lambda_{\tau -1}=i)}.$$

Thus, the MAP estimation of the MFR operating mode at time *t* is:

(16) $${\hat{\lambda }}_{MAP}=\arg\left(\underset{1\leq i\leq |\lambda |}{\max}\left(p(\lambda_{t}=i|h_{t})\right)\right).$$

The transition probabilities are not used in this algorithm. Physically, this is because the transitions of operating modes are dynamically judged via accumulating the predictive states *p*(*o_τ_*|*h_τ_*_−1_, *λ_τ_*_−1_ = *i*) instead of the fixed transition probabilities. The calculation of the predictive state is based on the nature of PSR, which expresses the current state by the distribution of future states. Without the requirement of fixed transition probabilities, the proposed approach is easier for application.

### 3.2. Fast Prediction for MFR Signals

Regarding the prediction of MFR signals, few studies are reported in public literatures. While PSR model can be naturally used as a predictor [30], the exact prediction probabilities of the future observations are available by making use of the property of linear PSR. This normal prediction algorithm shows the advantage of PSR in analysing MFR behaviours, but it remains to be improved in computational complexity especially for the high-dimensioned PSR model of MFR. In this section, a fast prediction algorithm is proposed with some engineering approximations. It is able to achieve good performance and decrease computational complexity significantly.

Firstly, the normal algorithm supported by linear PSR is briefly presented. The observation *k* steps ahead is predicted by considering the probabilities of all possible observation combinations from *t* to *t* + *k* − 1. Inputting these probabilities into the total probability formula, the probability of *o_t+k_* is estimated as:

(17) $$\begin{array}{ll} p(o_{t+k}|h_{t}) & =\sum_{i=1}^{\left|\lambda \right|}\left[p\left(o_{t+k}|h_{t},\lambda_{t}=i\right)\cdot p\left(\lambda_{t}=i|h_{t}\right)\right]\\ & =\sum_{i=1}^{\left|\lambda \right|}\left[\sum_{o_{t+k-1}o_{t+k-2}\cdots o_{t+1}\in O^{k-1}}p\left(o_{t+k}o_{t+k-1}\cdots o_{t+1}|h_{t},\lambda_{t}=i\right)\cdot p\left(\lambda_{t}=i|h_{t}\right)\right] \end{array}.$$

As mentioned above, the MFR mode for single target usually remains unchanged within a short time. Thus, it can be assumed that *λ_t_=λ_t+_*_1_*=…=λ_t+k_* for a small *k*. So with the same observation *o*, the parameters of **M**_o|*λ*_, as well as **m**_o|*λ*_, are the same for *λ_t_* to *λ_t+k_*. According to the property of linear PSR, for each possible combination of *o_t_*_+1_*o_t_*_+2_…*o_t_*_+*κ*−1_:

(18) $$p\left(o_{t+k}o_{t+k-1}\cdots o_{t+1}|h_{t},\lambda_{t}=i\right)=p^{T}\left(Q|h_{t},\lambda_{t}=i\right)\cdot M_{o_{t+1}|\lambda_{t}}M_{o_{t+2}|\lambda_{t}}\cdots M_{o_{t+k-1}|\lambda_{t}}m_{o_{t+k}|\lambda_{t}}.$$

Thus:

(19) $$\begin{array}{ll} p(o_{t+k}|h_{t}) & =\sum_{i=1}^{\left|\lambda \right|}\left[\sum_{o_{t+k-1}o_{t+k-2}\cdots o_{t+1}\in O^{k-1}}\left(p^{T}\left(Q|h_{t},\lambda_{t}=i\right)\cdot M_{o_{t+1}|\lambda_{t}}M_{o_{t+2}|\lambda_{t}}\cdots M_{o_{t+k-1}|\lambda_{t}}m_{o_{t+k}|\lambda_{t}}\right)\cdot p\left(\lambda_{t}=i|h_{t}\right)\right]\\ & =\sum_{i=1}^{\left|\lambda \right|}\left[p^{T}\left(Q|h_{t},\lambda_{t}=i\right)\cdot \sum_{o_{t+k-1}o_{t+k-2}\cdots o_{t+1}\in O^{k-1}}\left(M_{o_{t+1}|\lambda_{t}}M_{o_{t+2}|\lambda_{t}}\cdots M_{o_{t+k-1}|\lambda_{t}}\right)\cdot m_{o_{t+k}|\lambda_{t}}\cdot p\left(\lambda_{t}=i|h_{t}\right)\right]\\ & =\sum_{i=1}^{\left|\lambda \right|}\left[p^{T}\left(Q|h_{t},\lambda_{t}=i\right)\cdot {\left(\sum_{o\in O}M_{o|\lambda_{t}}\right)}^{k-1}\cdot m_{o_{t+k}|\lambda_{t}}\cdot p\left(\lambda_{t}=i|h_{t}\right)\right] \end{array}.$$

For the dimension of the observation space *|O|*, the number of operating modes |*λ*|, the number of core events |*Q*| and the known model parameters of **M***_o_*, the computational complexity for *k*-step prediction is approximately *o*(*k*∙|*λ*|∙*|O|*∙|*Q*|^3^) according to [36]. This suggests that the complexity is most affected with |*Q*|, especially for the high-dimensioned PSR models of MFR.

Considering a novel thought with less computation, i.e., viewing the predicted observation of each step as a known condition that can be used in predicting for the next moment. Then the multi-step prediction will be yielded by recurring to the required step. Thus, the large amount of computation caused by the uncertainty of the future observations can be avoided. The PSR-based fast prediction algorithm is presented as follows.

**Step 1:** Initialization, the single-step prediction: According to the distribution of operating modes, for each possible observation *o_t_* ∈ *O*, the single-step prediction probability is calculated as:

(20) $$p(o_{t+1}|h_{t})=\sum_{i=1}^{\left|\lambda \right|}\left[p(o_{t+1}|h_{t},\lambda_{t}=i)\cdot p(\lambda_{t}=i|h_{t})\right],$$

where *p*(*o_t_*_+1_|*h_t_*, *λ_t_* = *i*) is obtained from Equation (11), while *p*(*λ_t_* = *i|h_t_*) is obtained from Equation (15). Thus, the result of single-step prediction is:

(21) $${\left({\hat{o}}_{t+1}\right)}_{MAP}=\arg\left(\underset{o_{t+1}\in O}{\max}\left(p(o_{t+1}|h_{t})\right)\right).$$

Then, according to Equations (5) and (7), for any operating mode *λ_t_* = *i*:

(22) $$p^{T}\left(Q|h_{t}{\hat{o}}_{t+1},\lambda_{t}=i\right)=\frac{p^{T}\left(Q|h_{t},\lambda_{t}=i\right)M_{{\hat{o}}_{t+1}|\lambda_{t}}}{p^{T}\left(Q|h_{t},\lambda_{t}=i\right)m_{{\hat{o}}_{t+1}|\lambda_{t}}},\;i=1,2,\cdots ,\left|\lambda \right|,$$

(23) $$p\left({\hat{o}}_{t+1}|h_{t},\lambda_{t}=i\right)=p^{T}\left(Q|h_{t},\lambda_{t}=i\right)\cdot m_{{\hat{o}}_{t+1}|\lambda_{t}},\;i=1,2,\cdots ,\left|\lambda \right|.$$

**Step 2:** *k*-step ahead iterative prediction: The predicted observation is used in predicting for the next moment and recurring to the required step.

For the former *k* − 1 predicted observations ${\hat{o}}_{t+1}{\hat{o}}_{t+2}\cdots {\hat{o}}_{t+k-1}$ and the same assumption *λ_t_* = *λ_t+_*_1_ = … = *λ_t+k_*:

(24) $$p^{T}\left(Q|h_{t}{\hat{o}}_{t+1}{\hat{o}}_{t+2}\cdots {\hat{o}}_{t+k-1},\lambda_{t}=i\right)=\frac{p^{T}\left(Q|h_{t}{\hat{o}}_{t+1}{\hat{o}}_{t+2}\cdots {\hat{o}}_{t+k-2},\lambda_{t}=i\right)M_{{\hat{o}}_{t+k-1}|\lambda_{t}}}{p^{T}\left(Q|h_{t}{\hat{o}}_{t+1}{\hat{o}}_{t+2}\cdots {\hat{o}}_{t+k-2},\lambda_{t}=i\right)m_{{\hat{o}}_{t+k-1}|\lambda_{t}}},\;i=1,2,\cdots ,\left|\lambda \right|,$$

(25) $$p\left(o_{t+k}|h_{t}{\hat{o}}_{t+1}{\hat{o}}_{t+2}\cdots {\hat{o}}_{t+k-1},\lambda_{t}=i\right)=p^{T}\left(Q|h_{t}{\hat{o}}_{t+1}{\hat{o}}_{t+2}\cdots {\hat{o}}_{t+k-1},\lambda_{t}=i\right)\cdot m_{{\hat{o}}_{t+k}|\lambda_{t}},\;i=1,2,\cdots ,\left|\lambda \right|.$$

Therefore:

(26) $$p\left(o_{t+k}|h_{t}{\hat{o}}_{t+1}{\hat{o}}_{t+2}\cdots {\hat{o}}_{t+k-1}\right)=\sum_{\lambda_{t}=i}^{\left|\lambda \right|}\left[p\left(o_{t+k}|h_{t}{\hat{o}}_{t+1}{\hat{o}}_{t+2}\cdots {\hat{o}}_{t+k-1},\lambda_{t}=i\right)\cdot p\left(\lambda_{t}=i|h_{t}{\hat{o}}_{t+1}{\hat{o}}_{t+2}\cdots {\hat{o}}_{t+k-1}\right)\right].$$

For the factor $p\left(\lambda_{t}=i|h_{t}{\hat{o}}_{t+1}{\hat{o}}_{t+2}\cdots {\hat{o}}_{t+k-1}\right)$, the symbol *λ_t_* represents the operating mode at time *t*, which is only related to the history before *t*, rather than the predicted future observations ${\hat{o}}_{t+1}{\hat{o}}_{t+2}\cdots {\hat{o}}_{t+k-1}$. Then:

(27) $$p\left({\hat{o}}_{t+1}{\hat{o}}_{t+2}\cdots {\hat{o}}_{t+k-1}o_{t+k}|h_{t}\right)=\prod_{\kappa =2}^{k}p\left(o_{t+\kappa }|h_{t}{\hat{o}}_{t+1}{\hat{o}}_{t+2}\cdots {\hat{o}}_{t+\kappa -1}\right)\cdot p\left({\hat{o}}_{t+1}|h_{t}\right).$$

Thus:

(28) $$p(o_{t+k}|h_{t}{\hat{o}}_{t+1}{\hat{o}}_{t+2}\cdots {\hat{o}}_{t+k-1})=\frac{p\left({\hat{o}}_{t+1}{\hat{o}}_{t+2}\cdots {\hat{o}}_{t+k-1}o_{t+k}|h_{t}\right)}{\sum_{o_{t+k}\in O}p\left({\hat{o}}_{t+1}{\hat{o}}_{t+2}\cdots {\hat{o}}_{t+k-1}o_{t+k}|h_{t}\right)},$$

yielding:

(29) $${\left({\hat{o}}_{t+k}\right)}_{MAP}=\arg\left(\underset{o_{t+k}\in O}{\max}\left(p(o_{t+k}|h_{t}{\hat{o}}_{t+1}{\hat{o}}_{t+2}\cdots {\hat{o}}_{t+k-1})\right)\right).$$

The multi-step prediction of MFR signals is to be yielded by iterating from Equations (24) to (29). The computational complexity of the fast algorithm is analysed to be *o*(*k*∙|*λ*|∙*|O|*∙|*Q*|^2^), which is square with *|Q|* and lower than the normal algorithm. Although there are more steps in the novel algorithm, each of the steps has certain input and simple calculation. Therefore, this prediction algorithm has the advantages of low complexity compared with the normal algorithm. It is able to achieve fast prediction with less limitation of the model dimension and prediction steps, making it better suited for practical application.

## 4. Simulations

### 4.1. Simulation Settings

**Figure 3:**
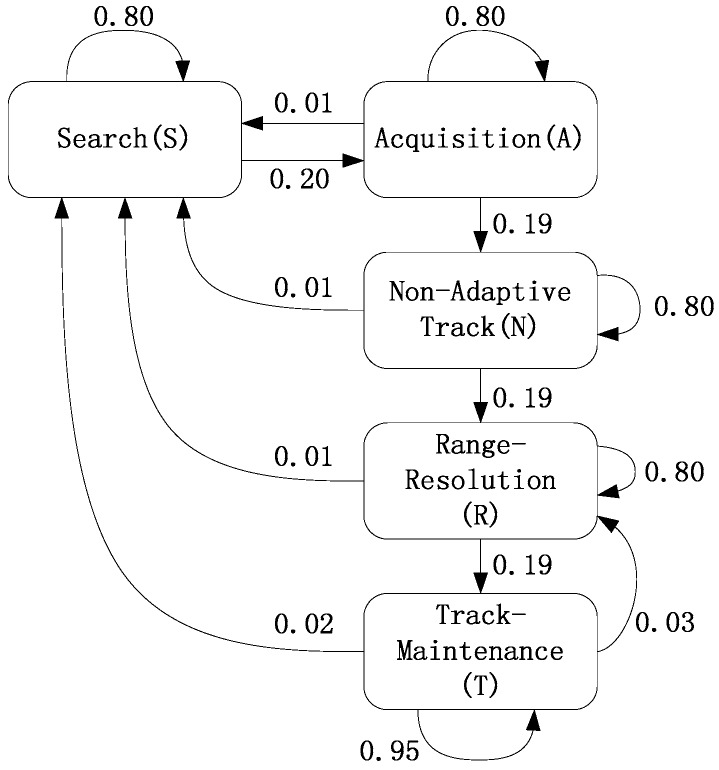
Operating mode transition of the MFR.

Taking the “Mercury” radar given in [18,21,22], which has five operating modes, denoted as search, acquisition, non-adaptive track, range resolution and track maintenance, for example. The mode transition of the MFR is shown in Figure 3, where the values denote the probabilities of corresponding mode transitions.

The list of MFR phrase combinations according to its operating modes is shown in Table 1. It can be seen that, each phrase of this MFR is a four-word sequence. One phrase may be shared in multiple operating modes, such as ‘*w*_1_*w*_6_*w*_6_*w*_6_’ and ‘*w*_6_*w*_6_*w*_6_*w*_6_’.

The data for simulation is generated as follows. For each PSR model of one operating mode, the training sequence is connected by 500 phrases. For generality, all the phrases belonging to this mode establish the cycling template with uniform random distribution. The template repeats with fixed cycling period to simulate the regular characteristics of MFR signals. In total, 10% of the radar words are corrupted to simulate mismatched words, which will lead to higher phrase error rate for the 4-word-length phrases. The test sequence contains 500 phrases with 10% mismatched words and simulates the entire process of target detection, acquisition, tracking and missing, cyclically employing the corresponding templates. With these settings, the collection of words *W* = {*w*_1_, *w*_2_, …, *w*_9_}, the observation space *O* = *W*^4^ and, thus, *|O|* = 9^4^; |*Q*| is the number of core events for each mode; the number of operating modes |*λ*| = 5.

In the simulations, the PSR-based MFR models are trained by using the training sequences of the five operating modes; MFR behaviour recognition and prediction are based on the pre-trained PSR framework, via the algorithms proposed in this paper.

### 4.2. Results

#### 4.2.1. Simulations of Operating Mode Recognition

**Figure 4:**
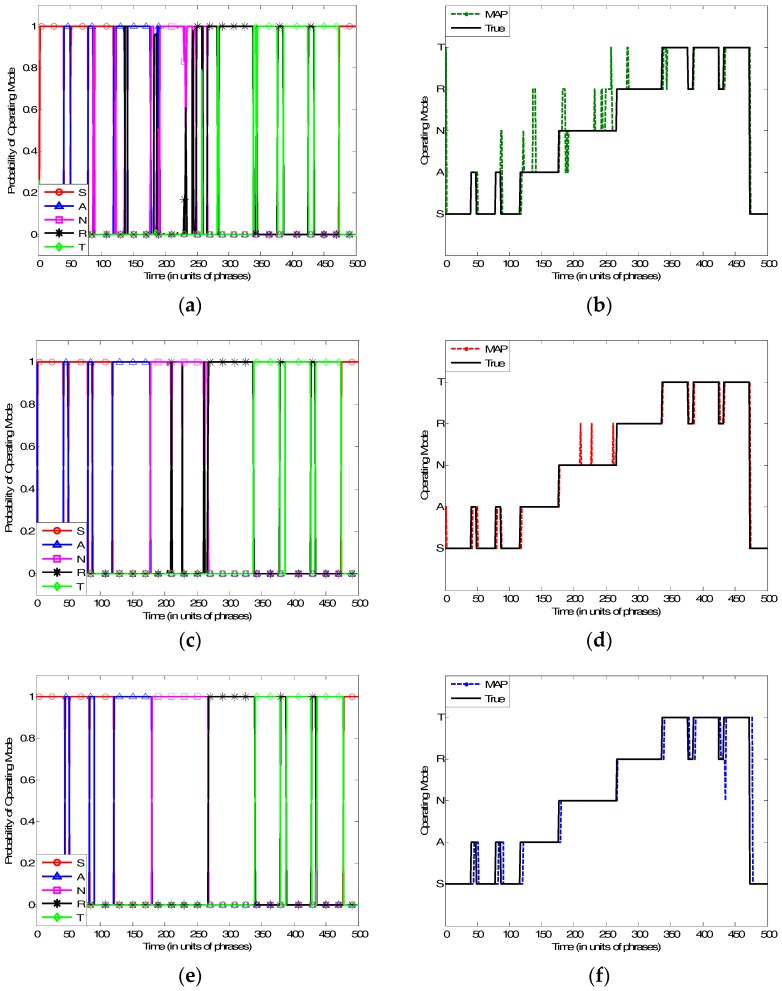
Recognition for MFR operating modes based on three recognition algorithms. (**a**) Probability distribution estimation based on HMM; (**b**) recognition based on HMM; (**c**) probability distribution estimation based on PSR and grid-filter; (**d**) recognition based on PSR and grid-filter; (**e**) probability distribution estimation based on PSR and proposed method; and (**f**) recognition based on PSR and proposed method.

The operating mode recognition results yielded via one HMM-based method and two PSR-based methods are presented in Figure 4.

Comparing with the results in Figure 4d,f which are yielded via the PSR-based methods, the dashed line in Figure 4b frequently deviates from the solid line, suggesting that the PSR-based methods significantly outperform the HMM-based method in MFR mode recognition. With the PSR model, the mode distribution yielded by grid-filter-based and the proposed method are shown, respectively, in Figure 4c,e. The figures indicate that the highest probability, which represents the recognized mode, is close to 1 at each moment. This suggests that the results yielded via both of these two algorithms are of high credibility.

As shown in Figure 4d,f, there are recognition errors in the results for both the two PSR-based algorithms. However, comparing the result lines with the true mode lines, the types of their errors are totally different. Errors in Figure 4d of Grid-filter-based method are all “misjudgements”, meaning that outputting a baseless wrong result despite a correction latter. While the errors in Figure 4f for the proposed method are mostly like “inertance”, which means remaining the original mode for a while after mode transitions. For ELINT systems, the cost of “misjudgements” is much higher than the latter, because the countermeasure decision requires reliable recognition results of MFR modes. From this point of view, the proposed method performs better under battlefield background.

**Figure 5:**
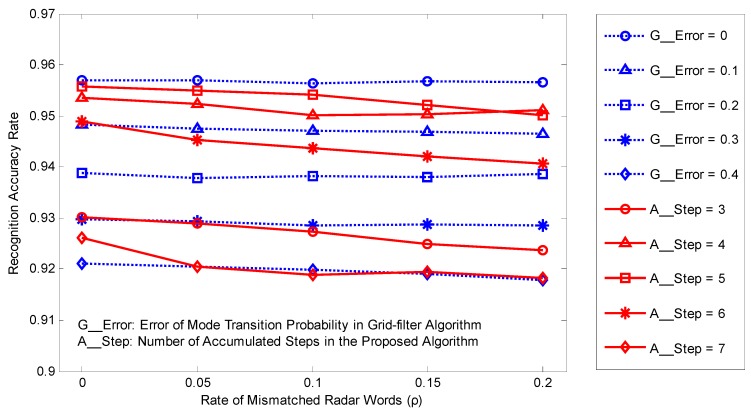
Recognition rate for the two mode recognition algorithms versus the impact factors.

The recognition performances of the two PSR-based algorithms are compared below, where the rate *ρ* of mismatched words in training sequences changes within 0~0.2. The average error of mode transition probabilities changes within 0~0.4 for the grid-filter and the number of accumulated steps ranges from 3 to 7 for the novel method. The results are shown in Figure 5.

The positions of the dotted lines in Figure 5 suggest that the recognition rate of the grid-filter-based algorithm decreases with increasing error of transition probability, while the solid lines representing the proposed method shows that with increasing accumulated steps, the performance gets better first, and then becomes worse, reaching the top when the number of accumulated steps is 5. This is because the fewer the accumulated steps for mode recognition, the less information is used; however, when the number of accumulated steps is too large, the recognition rate will be limited by the delayed recognition of changing modes. Moreover, the recognition rate changes quite slightly with different *ρ*, suggesting that both the algorithms are not sensitive to errors in the training sequences, which attests to the algorithms’ robustness.

Comparing the dotted curves with the solid curves on the top, it can be found that, with a proper number of accumulated steps, the novel algorithm is able to perform quite close to the grid-filter-based algorithm with accurate mode transition probabilities. The optimal accumulated steps can be obtained empirically from historical data. The novel method requires less prior information, which makes it suitable for practical application. In particular, if the timeliness is not strongly requited, the advantage of the proposed method is further highlighted.

#### 4.2.2. Simulations of Multi-Step Prediction

**Figure 6:**
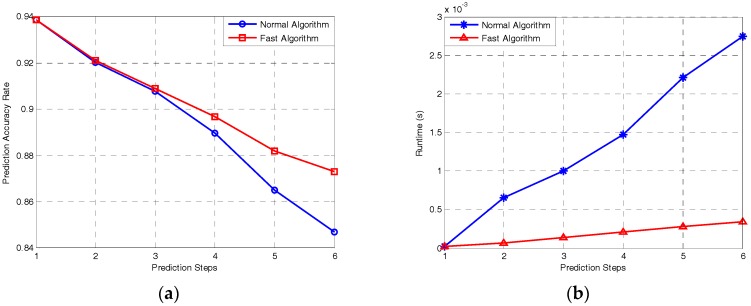
Prediction accuracy and efficiency of the normal algorithm and fast algorithm. (**a**) Prediction rate versus the number of prediction steps; and (**b**) runtime versus the number of prediction steps.

In Figure 6a, the prediction accuracy is measured based on the error rate of radar phrases, compared with the signal sequence that the MFR actually emits. The runtime of MATLAB (MathWorks, Natick, MA, USA) for the two algorithms is plotted in Figure 6b.

Figure 6a shows the prediction accuracy based on the two algorithms. As the trends of the curves present, the accuracy decreases generally with increasing prediction steps. This also suggests, unexpectedly, that the fast algorithm outperforms the normal algorithm. We analyse the data and discover that this is because the input of the normal algorithm is the state sequence with probability distribution, and that all the error of the intermediate results is accumulated continually with the increasing number of prediction steps. Conversely, in the fast algorithm, the error will be generally reset to zero after the MAP estimation; thus, the performance is even better than that of the other, in particular for high-step prediction.

The runtime of MATLAB for these two algorithms are plotted in Figure 6b. The tendency of the simulation time cost is approximately linear with the number of prediction steps, which attests to the validity of the computational complexity we inferred in Section 3.2. The runtime of the normal algorithm increases more rapidly comparing with that of the fast algorithm, which suggests that the proposed algorithm is more efficient.

In conclusion, the fast algorithm outperforms the normal algorithm with respect to both accuracy and efficiency, being able to achieve fast and accurate prediction of MFR signals multiple steps ahead.

## 5. Conclusions

This paper focuses on the topic of MFR behaviour recognition and prediction. Considering the flexibility and variety of MFR waveforms, the PSR is introduced due to its advantages in dynamical system expression and model training. Based on the PSR-based framework, the existing approaches are improved to be more suitable for application.

For the estimation of MFR modes, the common grid-filter-based methods require the mode transition probabilities as input data, which are unlikely to be obtained. We propose a novel algorithm for MAP estimation, judging the mode transitions by accumulating the predictive states instead of utilizing the fixed transition probabilities. Then, based on a novel iteration thought, a fast prediction algorithm is proposed. Compared with the normal prediction approach, the fast algorithm performs better with respect to both accuracy and efficiency when dealing with the high-dimensioned PSR model of MFR.

Simulation results suggest that the algorithms proposed for MFR mode recognition and signal prediction both have good performance. With the novel approach, the behaviours of MFR can be accurately recognized and predicted, which make it possible to dynamically analyse the action regulations and schedule schemes of MFRs and to achieve adaptive RCM in support of cognitive electronic warfare.

## Figures and Tables

**Table 1:** Phrases of the “Mercury” radar for each operating mode.

Mode		Phrases	Mode	Phrases
Search (S)	4-Word Search	[*w*_1_*w*_2_*w*_4_*w*_5_]	Track-Maintenance (T)	[*w*_1_*w*_7_*w*_7_*w*_7_]
		[*w*_2_*w*_4_*w*_5_*w*_1_]		[*w*_2_*w*_7_*w*_7_*w*_7_]
		[*w*_4_*w*_5_*w*_1_*w*_2_]		[*w*_3_*w*_7_*w*_7_*w*_7_]
		[*w*_5_*w*_1_*w*_2_*w*_4_]		[*w*_4_*w*_7_*w*_7_*w*_7_]
	3-Word Search	[*w*_1_*w*_3_*w*_5_*w*_1_]		[*w*_5_*w*_7_*w*_7_*w*_7_]
		[*w*_3_*w*_5_*w*_1_*w*_3_]		[*w*_6_*w*_7_*w*_7_*w*_7_]
		[*w*_5_*w*_1_*w*_3_*w*_5_]		[*w*_1_*w*_8_*w*_8_*w*_8_]
Acquisition (A)		[*w*_1_*w*_1_*w*_1_*w*_1_]		[*w*_2_*w*_8_*w*_8_*w*_8_]
		[*w*_2_*w*_2_*w*_2_*w*_2_]		[*w*_3_*w*_8_*w*_8_*w*_8_]
		[*w*_3_*w*_3_*w*_3_*w*_3_]		[*w*_4_*w*_8_*w*_8_*w*_8_]
		[*w*_4_*w*_4_*w*_4_*w*_4_]		[*w*_5_*w*_8_*w*_8_*w*_8_]
		[*w*_5_*w*_5_*w*_5_*w*_5_]		[*w*_6_*w*_8_*w*_8_*w*_8_]
Non-Adaptive Track (N) or Track-Maintenance (T)		[*w*_1_*w*_6_*w*_6_*w*_6_]		[*w*_1_*w*_9_*w*_9_*w*_9_]
		[*w*_2_*w*_6_*w*_6_*w*_6_]		[*w*_2_*w*_9_*w*_9_*w*_9_]
		[*w*_3_*w*_6_*w*_6_*w*_6_]		[*w*_3_*w*_9_*w*_9_*w*_9_]
		[*w*_4_*w*_6_*w*_6_*w*_6_]		[*w*_4_*w*_9_*w*_9_*w*_9_]
		[*w*_5_*w*_6_*w*_6_*w*_6_]		[*w*_5_*w*_9_*w*_9_*w*_9_]
Range-Resolution (R)		[*w*_7_*w*_6_*w*_6_*w*_6_]		[*w*_6_*w*_9_*w*_9_*w*_9_]
		[*w*_8_*w*_6_*w*_6_*w*_6_]		[*w*_7_*w*_7_*w*_7_*w*_7_]
		[*w*_9_*w*_6_*w*_6_*w*_6_]		[*w*_8_*w*_8_*w*_8_*w*_8_]
A or N or T		[*w*_6_*w*_6_*w*_6_*w*_6_]		[*w*_9_*w*_9_*w*_9_*w*_9_]
